# The Influence of Prior Hyperthyroidism on Euthyroid Graves' Ophthalmopathy

**DOI:** 10.1155/2014/426898

**Published:** 2014-06-22

**Authors:** Karolien Termote, Brigitte Decallonne, Ilse Mombaerts

**Affiliations:** ^1^Orbital Clinic, Department of Ophthalmology, University Hospitals Leuven, Kapucijnenvoer 33, 3000 Leuven, Belgium; ^2^Department of Endocrinology, University Hospitals Leuven, Herestraat 49, 3000 Leuven, Belgium

## Abstract

*Background*. To investigate the influence of previous exposure to elevated thyroid hormones in euthyroid Graves' ophthalmopathy. *Design*. Retrospective, observational case series in university setting Median follow-up of 1 year with ranges of 0,8–7,6 years. Study performance of 10 years. *Participants*. We reviewed the clinical records of 731 Graves' ophthalmopathy patients. There were 88 (12%) patients with onset of Graves' ophthalmopathy during euthyroidism: 37 (5%) patients had ophthalmopathy without known history of thyroid dysfunction (group A) and 51 patients (6%) had onset of ophthalmopathy 6 months or more euthyroid after completion of antithyroid therapy (group B). *Main Outcome Measures*. Graves' ophthalmopathy was graded using the EUGOGO severity criteria. Unilaterality was investigated. TSH receptor antibody and thyroid peroxidase antibody were measured as markers of Graves' disease. *Results*. Group A had more often a normal ocular motility (46%) and less proptosis (14 ± 4 mm) compared to group B (22%, 16 ± 4 mm) (*P* = 0.032 and 0.028, resp.). TSH receptor antibody was more frequently elevated in group B (94%) than in group A (17%) (*P* < 0.001). *Conclusion*. Patients with euthyroid Graves' ophthalmopathy present more often with ocular muscle restriction and proptosis when previously exposed to elevated thyroid hormones.

## 1. Introduction

Euthyroid Graves' ophthalmopathy (GO) is a GO without thyroid dysfunction and antithyroid treatment and is known to be a mild eye disease [1–3]. Measuring thyroid antibodies is considered to be helpful in the diagnosis. However, TSH-receptor (TRAb) and thyroid peroxidase antibodies (TPOAb) are negative in approximately 25% of euthyroid patients [1] and elevated in, respectively, 1-2% and 4–15% of healthy individuals [4–6]. In euthyroid GO cases without elevated antibodies, orbital imaging is indicated to support the diagnosis [7, 8]. It is believed that euthyroid GO is always associated with some degree of thyroid abnormality, as shown with second generation antibody assays [9, 10], thyroid scintigrams [11], and thyrotropin releasing hormone and T3 suppression testing [12–16].

Within the group of euthyroid GO patients, there is a subset of patients who have experienced thyroid dysfunction in the past. As severity of GO is linked to thyroid dysfunction, we hypothesized that previous exposure to elevated thyroid hormones in euthyroid GO patients may have an effect on the severity compared to those who never had had thyroid dysfunction [2, 3, 17]. The present study retrospectively analyses the clinical presentation and outcome of the different groups of euthyroid GO.

## 2. Methods

The medical records of all patients with the diagnosis of GO referred to the Orbital Clinic of the University Hospitals Leuven between June 1999 and June 2009, examined and treated by the same ophthalmologist (IM), were analysed retrospectively. This was performed respecting the guidelines of the Medical Ethics Committee of our institution.

The patients who developed GO under biochemical euthyroid conditions were divided into two groups: group A in the case of no reported history of thyroid dysfunction and group B in the case of a previous history of Graves' hyperthyroidism, in remission for more than 6 months after completion of antithyroid therapy (i.e. thiamazol, propylthiouracil, or treatment with radioactive iodine). Of the latter group, patients who developed hypothyroidism were substituted with levothyroxine. Excluded were all patients with less than 6 months of follow-up.

GO was diagnosed if one of the following findings were present: upper eyelid retraction (upper eyelid margin in the 12 o'clock position above the superior corneoscleral limbus in primary gaze); Hertel exophthalmometer reading >16 mm for women and >18 mm for men [18], anteriorly displaced globe on axial and coronal CT scan, or clinical proptosis when compared to predisease clinical photographs; or extraocular muscle restriction. Eyelid swelling was excluded from analysis, since, as a solitary finding, it is a weak argument in the diagnosis of euthyroid GO. Corneal dryness was also excluded from analysis, as it is a too variable finding, depending on the use of ocular lubricants. Axial and coronal orbital computed tomography scans were performed in all euthyroid GO patients at the first or second visit.

The GO was graded using the EUGOGO severity criteria and Mourits' clinical activity score (CAS) [19, 20]. The grading of NOSPECS class 4 was based on the limitation of ductions and the subjective diplopia score after Bahn and Gorman [21]. The CAS was graded out of 7 items during the first visit and out of 10 items from the second visit onwards. The highest severity and activity grade from at least 2 visits between a 2-month interval was used.

Thyroid function was biochemically analysed just prior to referral by the referring physician or at the time of the first visit at the orbital clinic and was repeated at each visit. Serum analysis included measurement of serum T4, T3, and TSH concentrations by electrochemiluminescence based assays (in our institution until November 2003 with the Immuno-1 analyzer (Bayer Diagnostics, Tarrytown, New York, USA), later with the Modular Analytics E (Roche Diagnostics, Basel, Switzerland)). Euthyroidism was defined as serum values of the thyroid hormones within the laboratory reference range, in combination with normal TSH values. Subnormal TSH levels were allowed in the secondary euthyroid group, since TSH-receptor-binding inhibitory immunoglobulins can cause a long lasting downregulation of TSH release despite normal T3 and T4 levels [22].

TSH-receptor antibody (TRAb) and thyroid peroxidase antibody (TPOAb) were measured as markers of Graves' disease (in our institution first with a radioimmunoassay (B.R.A.H.M.S. Diagnostica, Berlin, Germany), later with a electrochemiluminescence based assay, for TPOAb the Modular Analytics E (Roche Diagnostics, Basel, Switzerland) and for TRAb the TRAK human (B.R.A.H.M.S. Diagnostica, Berlin, Germany)) [23].

Statistical significance was based on the Mann-Whitney test for continuous variables and on the Fisher's exact test for dichotomized variables. The influence of gender, age, and smoking on significant differences between groups was studied using a logistic regression analysis. A *P* value of <0.050 was regarded as significant and of <0.010 as highly significant. Statistical evaluations were performed by SPSS software (Statistical Package for the Social Sciences; version 16.0, SPSS Inc. Headquarters, 233 S. Wacker Drive, 11th floor Chicago, Illinois 60606).

## 3. Results

From June 1999 to June 2009, 788 patients with GO were seen in one orbital center. Fifty-seven patients were excluded for insufficient laboratory findings (54) or insufficient follow-up (3). From the remaining 731 patients, 37 (5%) were included in group A and 41 (6%) in group B. All patients were Caucasian except one black male in group A and one Asian female in group B.  Table 1 outlines the patients' characteristics. The median age was higher in the secondary euthyroid group (*P* = 0.004).

The median follow-up was 1 year in both groups with a mean of 3 years in group A and 2 years in group B (*P* = 0.018). During follow-up, 3 (8%) patients of group A developed hyperthyroidism, at 1, 5, and 6 years, respectively. The median time interval between the onset of hyperthyroidism and the onset of GO in group B was 4 years (range 1–34 years). Relapse of hyperthyroidism occurred in 1 (2%) patient of group B, 11 months after the onset of GO. Hypothyroidism due to inadequate substitution therapy occurred in 1 (2%) patient of group B, 21 months after onset of GO.

Table 2 displays the clinical signs and severity of both GO groups. There was a unilateral presentation of GO in 32% of both groups. Unilaterality of euthyroid GO was not significantly correlated to other clinical characteristics. For instance there was no significant increase in upper eyelid retraction as solitary finding in unilateral GO. Of all the clinical GO signs, only the presence of ocular motility restriction and the amount of proptosis were statistically different (highly significant) between both groups, with group A being less affected. Group A presented significantly more often with normal eye motility (46%) compared to group B (22%) (*P* = 0.032). Using the EUGOGO criteria for GO severity, there was no statistically significant difference in severity among both groups.

Antibodies were determined in 97% (36/37) of the patients of group A and in 80% (33/41) of group B. The prevalence of positive TRAbs was significantly higher in group B (94%) compared to group A (17%) (*P* < 0.001) (Table 3). There was no significant correlation between the prevalence of TRAbs and the disease severity of euthyroid GO nor between the prevalence of TRAbs and the clinical presentation of euthyroid GO. The latter correlation was calculated for each subset of the NOSPECS score but was not significant for any parameter.

The treatment modalities of Graves' disease and GO are displayed in Table 4. Of the 33 patients of group B who received radioiodine, 27 (82%) received substitution therapy. All patients who received oral corticosteroid treatment for GO had the treatment prior to referral to us. The number of patients who underwent strabismus surgery was not significantly different among both groups.

## 4. Discussion

Our findings of a mild, often unilateral GO in euthyroid patients are in agreement with studies on euthyroid GO that deal mostly with patients without a dysthyroid past, labelled by us as group A [1, 3, 12]. This finding supports the theory that GO benefits from euthyroid conditions [2]. We found that a prior history of hyperthyroidism in euthyroid GO is associated with extraocular muscle restriction and proptosis. This is a new finding. The higher prevalence of muscle restriction and proptosis in group B, however, was not reflected in a higher amount of strabismus or orbital decompression surgery.

The onset of GO coincides with the onset of hyperthyroidism in 20–43% of patients [16, 24, 25]. GO precedes the diagnosis of hyperthyroidism in 14–28% of the cases and follows it in 28–57%, mainly within 18 months [16, 24, 25]. In true euthyroid GO, however, the orbital disease develops without a present or past hyperthyroidism. Repeated measurements of thyroid function are essential during the follow-up, since euthyroid GO can be the initial stage of thyroid disease. In this study the follow-up in group A was significantly longer than in group B with a mean of 3 and 2 years, respectively. This is probably because the examiner did not want to miss subsequent development of dysthyroidism in group A. The latter occurred in only 8% of the patients of group A.It is, however, possible that this is underestimated, as the patients may have transient undetected episodes of dysthyroidism in between the follow-up visits. Furthermore, the follow-up might be too short to detect late onsets of dysthyroidism. There was a long time interval between the development of thyroid and eye disease in group B (median of 4 and a maximum of 34 years). This suggests that previous exposure to elevated thyroid hormones may have a sustained effect on the eye.

The higher prevalence of extraocular muscle restriction and proptosis in group B compared to group A may reflect a delayed result of previous exposure to elevated thyroid hormones. However, it is unlikely that the eye disease takes as much as 4 years to manifest itself clinically. Previous studies have shown that only 5–8% of the hyperthyroid patients develop GO more than 3 and 4 years after onset of hyperthyroidism, whilst approximately 81–85% develop GO within 18 months before or after the onset of hyperthyroidism [16, 24]. Another likely explanation is a transient initially undiagnosed GO at time of the hyperthyroidism. It is believed that the majority of the patients with onset of Graves' disease develop some degree of GO, often subclinically, not noticed by the patient nor the treating physician [26]. In the latter case, reactivation of dysthyroid GO would be the preferred diagnosis rather than euthyroid GO with previous exposure to elevated thyroid hormones [27].

Several factors may have influenced the difference of clinical presentation of GO between both groups. Firstly, 80% of the patients of group B received radioiodine therapy, while—by definition—none of group A did. However all patients at risk for radioiodine-induced activation of GO, received oral corticosteroid prophylaxis during radioiodine treatment and adequate levothyroxine substitution afterwards. We feel that the influence of radioiodine was hence existent but minimal in this study. A second epidemiological factor that may have influenced our results is the older age in group B. GO tends to be more severe in older age, especially with a higher risk of optic neuropathy [28]. The potential relation between age and extraocular muscle restriction in GO is controversial in the literature [28, 29]. The older age in group B was, however, not reflected in a more severe GO with optic neuropathy. Disease severity is also higher in smokers, with more disease progression and poorer outcome of therapy [26]. The incidences of smoking in both euthyroid groups were, however, similar to those reported for GO in general, that is, approximately 40% [30].

The study design is a retrospective, observational case series, which has inherent disadvantages. Some patients received endocrinological follow-up in other hospitals and different laboratory analysis methods might have been used. Hence, the absolute values of the antibody levels could not be compared. Therefore we divided the patient groups into those with normal and those with elevated antibody levels. The diagnosis of euthyroid GO without elevated thyroid antibodies was based on the clinicoradiological picture. There was a higher prevalence of elevated TRAbs in group B. This may be attributed to the frequent use of radioiodine therapy that may be accompanied with an elevation of TRAbs [31]. The higher prevalence of elevated TRAbs in group B compared to group A was correlated with more proptosis and extraocular muscle involvement, however, not with eye disease severity. This is probably because the significant differences in clinical presentation between both groups were not large enough to assign higher NOSPECS severity grades to group B. Eckstein et al. found, however, that TRAbs can be predictors of disease severity in GO in general [31]. If elevated in euthyroid GO, TRAbs can also be the predictors for future development of hyperthyroidism [10]. This was not observed in our study.

Finally, since euthyroid unilateral GO patients without previous thyroid dysfunction are more often referred to a specialist center like our orbital clinic, there may be a selection bias. They are frequently mild cases who only need a confirmation of diagnosis.

In conclusion, this study shows a different clinical spectrum of euthyroid GO according to previous exposure to elevated thyroid hormones. We found euthyroid GO with previous exposure to elevated thyroid hormones to have more extraocular muscle involvement and proptosis, as well as more frequently elevated TRAbs, compared to euthyroid GO without previous thyroid dysfunction. As euthyroid GO can develop several years after previous dysthyroidism, a constant awareness of this late onset GO is warranted. A better understanding of the development of euthyroid GO can minimize the delay of diagnosis, which is one of the 5-year targets in the Amsterdam EUGOGO declaration [32].

## Figures and Tables

**Table 1:** Patients' details.

	Group A *N* = 37	Group B *N* = 41	*P* Value
Female/male (% female)	25/12 (68%)	35/6 (85%)	NS^†^
Age, median years (mean; range)	48 (46; 20–80)	53 (56; 28–83)	0.004
Smokers	16 (43%)	18 (44%)	NS^†^
Follow-up, median years (mean; range)	1 (3; 1–8)	1 (2; 1–7)	0.018
Familial incidence of GD^‡^	13 (35%)	13 (32%)	NS^†^
Familial incidence of GO^§^	4 (11%)	4 (10%)	NS^†^

Values are expressed as numbers, except as if indicated.

^†^NS = not significant.

^‡^GD = Graves' disease.

^§^GO = Graves' ophthalmopathy.

**Table 2:** Difference in clinical presentation and severity of euthyroid Graves' ophthalmopathy.

Clinical presentation	Group A (*N* = 37)	Group B (*N* = 41)	*P* Value
Unilateral disease	12 (32%)	13 (32%)	NS^†^
Time interval onset thyroid-eye disease, median years (mean; range)	—	4 (8; 1–34)	—
Clinical activity score, mean (±SD^‡^)	1 (±1)	2 (±1)	NS^†^
Upper eyelid retraction, mean (±SD^‡^)	1 mm (±1)	1 mm (±1)	NS^†^
Hertel reading, mean (±SD^‡^)	14 mm (±4)	16 mm (±4)	0.028
Extraocular muscle involvement			
Normal eye motility	17 (46%)	9 (22%)	0.032
NOSPECS class 4 grade a	16 (43%)	22 (54%)	NS^†^
NOSPECS class 4 grade b	4 (11%)	10 (24%)	NS^†^
Severity			
Mild	14 (38%)	12 (29%)	NS^†^
Moderate-to-severe	23 (62%)	28 (68%)	NS^†^
DON^§^	0 (0%)	1 (2%)	NS^†^

Values are expressed as numbers, except as if indicated.

^†^NS = not significant.

^‡^SD = standard deviation.

^§^DON = dysthyroid optic neuropathy.

**Table 3:** Prevalence of thyroid antibodies.

Thyroid antibodies	Group A (*N* = 36)	Group B (*N* = 33)	*P* Value
Elevated TRAb^†^	6 (17%)	31 (94%)	<0.001
Elevated TPOAb^‡^	12 (33%)	14 (42%)	NS^§^
Normal TRAb^†^ and TPOAb^‡^	22 (61%)	2 (6%)	<0.001

Values are expressed as numbers, except as if indicated.

^†^TRAb = TSH-receptor antibody.

^‡^TPOAb = thyroid peroxidase antibody.

^§^NS = not significant.

**Table 4:** Therapy for Graves' hyperthyroidism and Graves' ophthalmopathy.

Therapy	Group A (*N* = 37)	Group B (*N* = 41)	*P* Value
Smoking cessation	3/16 (19%)	4/18 (22%)	NS^†^
Antithyroid drugs	—	22 (54%)	—
Thyroidectomy	—	2 (5%)	—
Radioiodine	—	33 (80%)	—
Orbital irradiation	3 (8%)	0 (0%)	NS^†^
Corticosteroids^‡^	2 (5%)	2 (5%)	NS^†^
Orbital decompression	1 (3%)	5 (12%)	NS^†^
Strabismus surgery	7 (19%)	12 (29%)	NS^†^
Upper eyelid blepharoplasty	2 (5%)	4 (10%)	NS^†^
Upper eyelid lengthening surgery	6 (16%)	4 (10%)	NS^†^

Values are expressed as numbers, except as if indicated.

^†^NS = not significant.

^‡^For moderate-to-severe GO: methylprednisolon 64 mg/d orally, with taper over 2-3 months and for 1 patient in group B with dysthyroid optic neuropathy: methylprednisolon intravenously 6 × 1000 mg/d over 2 weeks, with taper orally.
